# Discovering the Molecular Determinants of Phaeobacter inhibens Susceptibility to Phaeobacter Phage MD18

**DOI:** 10.1128/mSphere.00898-20

**Published:** 2020-11-04

**Authors:** Guillaume Urtecho, Danielle E. Campbell, David M. Hershey, Fatima A. Hussain, Rachel J. Whitaker, George A. O’Toole

**Affiliations:** a Molecular Biology Interdepartmental Doctoral Program, University of California, Los Angeles, Los Angeles, California, USA; b Department of Microbiology, University of Illinois Urbana-Champaign, Urbana, Illinois, USA; c Department of Biochemistry and Molecular Biology, University of Chicago, Chicago, Illinois, USA; d Department of Civil and Environmental Engineering, Massachusetts Institute of Technology, Cambridge, Massachusetts, USA; e Carl R. Woese Institute for Genomic Biology, University of Illinois Urbana-Champaign, Urbana, Illinois, USA; f Department of Microbiology and Immunology, Geisel School of Medicine at Dartmouth, Hanover, New Hampshire, USA; Clemson University

**Keywords:** bacteriophage, BarSeq, ecology, microbial interactions, phage genomics

## Abstract

Bacteriophages have immense potential as antibiotic therapies and in genetic engineering. Understanding the mechanisms that bacteriophages implement to infect their hosts will allow researchers to manipulate these systems and adapt them to specific bacterial targets. In this study, we isolated a bacteriophage capable of infecting the marine alphaproteobacterium Phaeobacter inhibens and determined its mechanism of infection*. Phaeobacter virus MD18*, a novel species of bacteriophage isolated in Woods Hole, MA, exhibits potent lytic ability against P. inhibens and appears to be of the *Siphoviridae* morphotype. The genomic sequence of MD18 displayed significant similarity to another siphophage, the recently discovered Roseobacter phage DSS3P8, but genomic and phylogenetic analyses, assessing host range and a search of available metagenomes are all consistent with the conclusion that Phaeobacter phage MD18 is a novel lytic phage. We incubated MD18 with a library of barcoded *P. inhibens* transposon insertion mutants and identified 22 genes that appear to be required for phage predation of this host. Network analysis of these genes using genomic position, Gene Ontology (GO) term enrichment, and protein associations revealed that these genes are enriched for roles in assembly of a type IV pilus (T4P) and regulators of cellular morphology. Our results suggest that T4P serve as receptors for a novel marine virus that targets *P. inhibens.*

**IMPORTANCE** Bacteriophages are useful nonantibiotic therapeutics for bacterial infections as well as threats to industries utilizing bacterial agents. This study identified *Phaeobacter virus MD18*, a phage antagonist of Phaeobacter inhibens, a bacterium with promising use as a probiotic for aquatic farming industries. Genomic analysis suggested that Phaeobacter phage MD18 has evolved to enhance its replication in *P. inhibens* by adopting favorable tRNA genes as well as through genomic sequence adaptation to resemble host codon usage. Lastly, a high-throughput analysis of *P. inhibens* transposon insertion mutants identified genes that modulate host susceptibility to phage MD18 and implicated the type IV pilus as the likely receptor recognized for adsorption. This study marks the first characterization of the relationship between *P. inhibens* and an environmentally sampled phage, which informs our understanding of natural threats to the bacterium and may promote the development of novel phage technologies for genetic manipulation of this host.

## Abstract (precis)

Bacteriophages are useful nonantibiotic therapeutics for bacterial infections as well as threats to industries utilizing bacterial agents. This study identified *Phaeobacter virus MD18*, a phage antagonist of Phaeobacter inhibens, a bacterium with promising use as a probiotic for aquatic farming industries. Genomic analysis suggested that Phaeobacter phage MD18 has evolved to enhance its replication in *P. inhibens* by adopting favorable tRNA genes as well as through genomic sequence adaptation to resemble host codon usage. Lastly, a high-throughput analysis of *P. inhibens* transposon insertion mutants identified genes that modulate host susceptibility to phage MD18 and implicated the type IV pilus as the likely receptor recognized for adsorption. This study marks the first characterization of the relationship between *P. inhibens* and an environmentally sampled phage, which informs our understanding of natural threats to the bacterium and may promote the development of novel phage technologies for genetic manipulation of this host.

## INTRODUCTION

Viruses are the largest known reservoir of genetic diversity. As such, they have given rise to an incredible assortment of genetic tools and potential therapeutics used as sustainable substitutes for antibiotics (1, 2), targeted bacterial delivery systems (3), and bacterial engineering (4). However, the unique ecological relationships and infection mechanisms that have evolved in the wake of the phage-host arms race are diverse and complex, making thorough characterization necessary before leveraging these systems for therapeutic and genetic engineering approaches. Further studies of phage-host interactions are essential to understand and manipulate the mechanisms phages implement to infect their host.

Phaeobacter inhibens is a marine bacterium and member of the *Roseobacteraceae* family of *Alphaproteobacteria*. Organisms in this clade are found worldwide, especially near coastal waters (5, 6), and may constitute up to 30% of bacterial communities in the open ocean (7). Investigations of *Roseobacter* organisms in their environmental niche have revealed extensive associations with marine algae and the production of a diverse array of secondary metabolites (8, 9). P. inhibens is most known for the production of the antibiotic tropodithietic acid (TDA), which protects its natural algal symbiont from marine pathogens (9, 10). Due to this property, *P. inhibens* serves as a useful probiotic in oyster and other aquatic farms to prevent colonization by pathogenic *Vibrio* species (11, 12). Despite its importance in the aquatic farming industry, viral predators that target this microbe remain poorly understood. Characterizing phage-host interactions within this industrially relevant host will enable researchers to implement phage engineering approaches for this species, provide insight into the viral antagonists of *P. inhibens*, and develop strategies to prevent phage predation that may threaten industrial aquaculture.

In this study, we isolated a siphophage capable of infecting *P. inhibens* from an aquatic environment in Woods Hole, MA, and characterized this phage using transmission electron microscopy (TEM) and whole-genome sequencing. Based on the morphology and genomic comparison to other related phages, we found that this isolate represents a novel species of siphophage, which we name *Phaeobacter virus MD18*. Finally, we used a barcoded transposon insertion mutant library (i.e., BarSeq) to characterize the relationship between Phaeobacter phage MD18 and *P. inhibens*. We identified the type IV pilus system, the ChvI/ChvG two-component system, and regulators of cell division as key determinants of infection. This work characterizes the genetic basis of phage infection in an underexplored nonmodel host system.

## RESULTS

### Isolation and characterization of Phaeobacter phage MD18.

**FIG 1:**
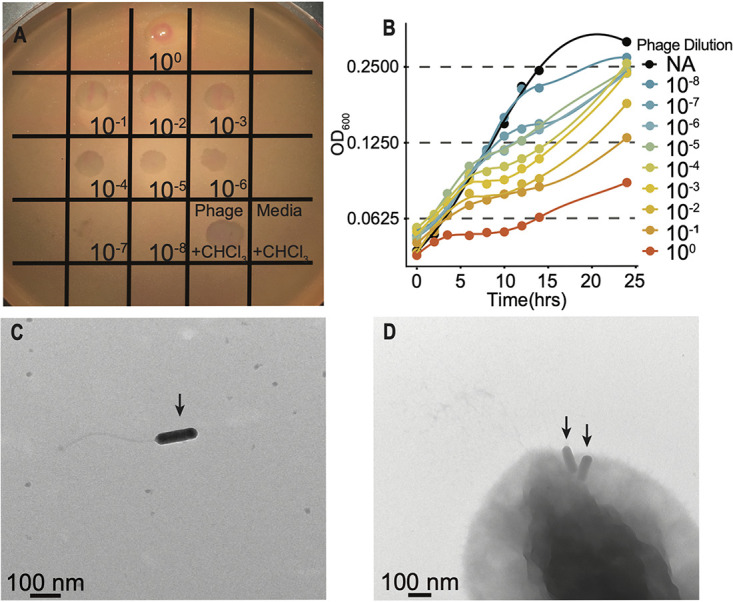
Characterization of phage MD18. (A) Host lysis after top agar spotting of Phaeobacter phage MD18 onto a lawn of *P. inhibens*. Approximately 5 plaques were detected in the 10^−7^ dilution. Given that 5 μl of the lysate was spotted here, this corresponds to a phage titer of ∼10^−10^ PFU/ml. Chloroform-treated control (+CHCl_3_), spotted here as the undiluted lysate, suggests that cellular predators are not responsible for plaque formation. Medium control also underwent chloroform treatment and did not cause lysis. (B) Growth of *P. inhibens* in the presence of the indicated titer of phage MD18, as monitored by optical density at 600 nm (OD_600_). NA, none added, is the no-phage control. Each line represents the average of three growth curves performed in a 96-well plate. See Materials and Methods for additional details. (C) Transmission electron microscopy (TEM) identified phage particles resembling *Siphoviridae*. (D) TEM image of phage MD18 (arrows) apparently adsorbed to the surface of *P. inhibens*. Phage particles are adjacent to apparent pilus-like structures. A scale bar is shown in panels C and D.

We obtained samples from 20 aquatic environments around Woods Hole, MA, to isolate a wild bacteriophage capable of infecting *P. inhibens*. We enriched for phages infecting *P. inhibens* by incubating filtered environmental samples together with exponentially growing liquid cultures of *P. inhibens* overnight. Then, we filtered the enrichment cultures and spotted each enrichment supernatant onto a lawn of *P. inhibens* grown on agar plates and monitored for the formation of plaques. Of the 20 environmental samples, only 1 sourced from a seashore environment produced a clear plaque, indicating the presence of a lytic phage. This enriched phage sample contained a high titer of PFU (∼10^10^ PFU/ml [Fig. 1A]) and repressed host growth in liquid culture at concentrations of the phage below the limit of detection by plaquing (<200 PFU/ml [Fig. 1B]). Growth inhibition occurred after incubation of the phage lysate with chloroform (Fig. 1A), confirming that predatory cellular microbes (e.g., *Bdellovibrio* spp.) were not responsible for the observed inhibition. Propagation of clear plaques indicated essentially complete bacterial host lysis and thus was likely the result of a lytic phage, henceforth referred to as Phaeobacter phage MD18.

Lastly, we explored the host range of MD18 by challenging another alphaproteobacterium, Caulobacter crescentus. MD18 was incapable of forming plaques on C. crescentus under conditions in which ΦCbK readily formed plaques (see Fig. S1 in the supplemental material), indicating some level of specificity to *P. inhibens* by MD18. Although relatively few small plaque-like structures did form when ΦCbK was spotted onto *P. inhibens*, the numbers of plaques were constant across different concentrations of ΦCbK, suggesting that these structures were not due to lysis by ΦCbK.

10.1128/mSphere.00898-20.1 FIG S1 Phaeobacter phage MD18 exhibits lytic specificity to *P. inhibens*. A dilution series of MD18 and C. crescentus phage ΦCbK were spotted on soft agar overlays containing *P. inhibens* (top) and C. crescentus (bottom). Phages demonstrated host-specific lytic potential. Download FIG S1, PDF file, 1.4 MB. Copyright © 2020 Urtecho et al. 2020 Urtecho et al. This content is distributed under the terms of the Creative Commons Attribution 4.0 International license.

To analyze phage MD18 morphology, we visualized filter-sterilized phage lysates by TEM. TEM revealed uniform phage particles with prolate heads roughly 100 nm in length, within a normal range for bacteriophages (13) (Fig. 1C). The long, flexible tail suggests that phage MD18 displays the *Siphoviridae* morphotype (14). Furthermore, we observed incidences of MD18 particles adsorbed to the surfaces of *P. inhibens* cells, positioned roughly at the bacterial cell pole (Fig. 1D). Together, these results suggest that MD18 is a potent lytic phage of the *Siphoviridae* morphotype that likely recognizes a structure on the *P. inhibens* cell surface.

### Genome sequence of Phaeobacter phage MD18 represents a novel species.

**FIG 2:**
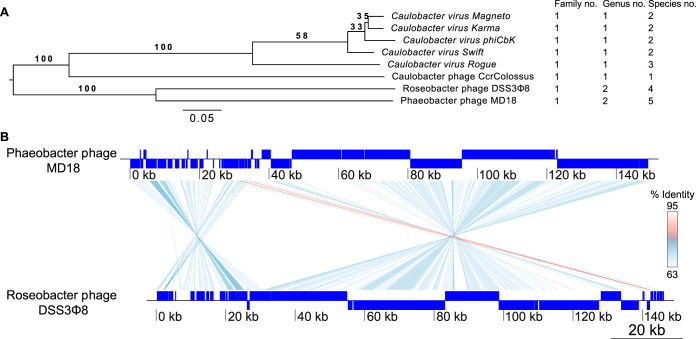
*Phaeobacter virus MD18* is a novel species. (A) Phylogenetic analysis of Phaeobacter phage MD18 and related phage isolates. The numbers above the branches are genome BLAST distance phylogeny pseudobootstrapped support values from 100 replications. The branch lengths of the resulting VICTOR trees are scaled in terms of the respective distance formula used. The OPTSIL clustering yielded five species, two genera, and one family. (B) Genome-wide comparison between Phaeobacter phage MD18 and Roseobacter phage DSS3P8. Connected regions indicate areas of significant sequence identity between these genomes according to BLAST. Color scale is an approximation.

We further characterized phage MD18 by performing whole-genome shotgun sequencing. We assembled a single circular contig, which was 149,262 bp in length. The resolution of a singular contig suggests that the enriched phage isolate contained a singular phage species rather than a mixed phage population. A BLAST search of this assembly revealed notable sequence similarity to the recently discovered Roseobacter phage DSS3P8 (Fig. S2), which is also of the *Siphoviridae* morphotype and a close relative of the CbK-like phages which infect Caulobacter crescentus (15, 16). To clarify the relationship of MD18 and other phages, we performed a phylogenetic analysis encompassing MD18, DSS3P8, and six Cbk-like phages (17) using the Virus Classification and Tree Building Online Resource (VICTOR) (Fig. S3). The phylogenetic tree with the highest support suggests that Phaeobacter phage MD18 is within the same family as the CbK phages and shares a genus with Roseobacter phage DSS3P8 yet is a unique species (Fig. 2A). We then performed a genome-wide comparison between MD18 and DSS3P8 to further delineate these genomes (Fig. 2B). BLAST comparison identified significant regions of homology between these genomes spanning 48% of the MD18 genome with an average of 74.1% identity. Notably, this analysis revealed large-scale genomic rearrangements that likely occurred during the speciation of these phages. In light of the classification guidelines established by the International Committee on Viral Taxonomy’s Bacterial and Archaeal Viruses Subcommittee (18), Phaeobacter phage MD18 represents a novel species.

10.1128/mSphere.00898-20.2 FIG S2 Genomic comparison between Phaeobacter phage MD18 genome and Roseobacter phage DSS3P8. Sequence alignments of Phaeobacter phage MD18 compared to the nearest genomic match, Roseobacter phage DSS3P8, were visualized using the BLAST Ring Image Generator (BRIG) (N.-F. Alikhan, N. K. Petty, N. L. Ben Zakour, and S. A. Beatson, BMC Genomics 12:402, 2011, https://doi.org/10.1186/1471-2164-12-402). Highlighted regions indicate sections exhibiting over 50% sequence similarity with Roseobacter phage DSS3P8. Despite sequence similarity, gene order appears to differ between these two phages (Fig. 2B). Download FIG S2, PDF file, 0.2 MB. Copyright © 2020 Urtecho et al. 2020 Urtecho et al. This content is distributed under the terms of the Creative Commons Attribution 4.0 International license.

10.1128/mSphere.00898-20.3 FIG S3 Phylogenetic analysis of *Phaeobacter virus MD18* and related phages. Phylogenomic genome BLAST distance phylogeny trees inferred using the formulas D0, D4, and D6 and yielding average support of 55%, 59%, and 65%, respectively. The numbers above branches are GBDP pseudobootstrap support values from 100 replications. The branch lengths of the resulting VICTOR trees are scaled in terms of the respective distance formula used. The OPTSIL clustering yielded seven (D0), eight (D4), and five (D6) species clusters. At the genus level, two (D0), five (D4), and two (D6) clusters resulted. The number of clusters determined at the family level were one (D0), five (D4), and one (D6). Download FIG S3, PDF file, 0.2 MB. Copyright © 2020 Urtecho et al. 2020 Urtecho et al. This content is distributed under the terms of the Creative Commons Attribution 4.0 International license.

Finally, we assessed whether MD18 had been previously identified in metagenomes. We used BLAST to search the MD18 genome against 27,346 marine virome contigs assembled from 78 marine viromes (19). This search uncovered a single significant match of 29 bp. Thus, we conclude that *Phaeobacter virus MD18* corresponds to a unique species that has not previously been identified.

### Genome content of *Phaeobacter virus MD18* and evidence of host adaptation.

We annotated the genome using RAST (20, 21) and identified 257 putative genes (Table S1), of which 215 represented “hypothetical proteins.” Notable identified genes include a RecD-like DNA helicase and a DNA polymerase III ε subunit, which are likely components of the phage replication system, as well as an *N*-acetylmuramoyl-l-alanine amidase, which have been demonstrated to facilitate bacterial cell wall degradation and cell lysis (22, 23).

10.1128/mSphere.00898-20.5 TABLE S1 Annotated features in Phaeobacter phage MD18 genome by RAST. Download Table S1, XLSX file, 0.1 MB. Copyright © 2020 Urtecho et al. 2020 Urtecho et al. This content is distributed under the terms of the Creative Commons Attribution 4.0 International license.

**FIG 3:**
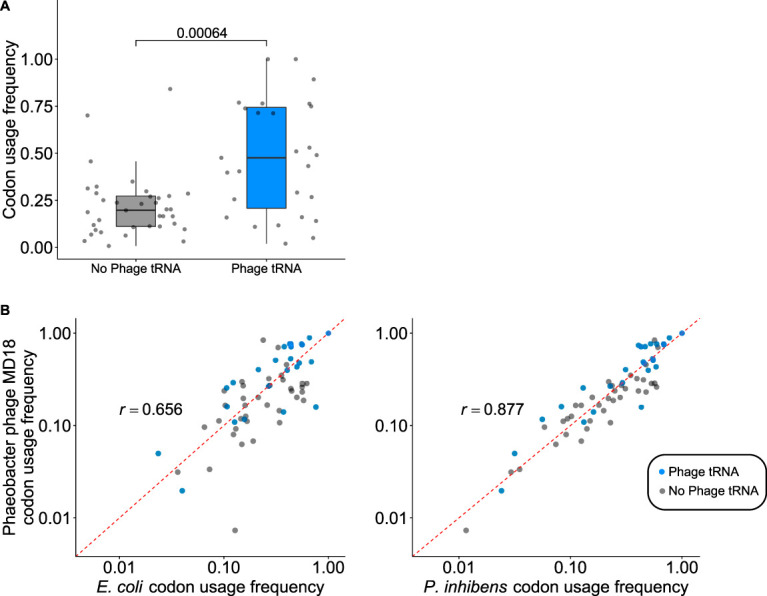
Bacteriophage tRNA genes show adaptation to host and phage genomes. (A) Codons corresponding to phage-encoded tRNA genes are significantly enriched in the Phaeobacter phage MD18 genome compared to codons without a corresponding phage tRNA gene (*P = *6.4 × 10^−5^, Wilcox rank sum test). (B) Codon usage frequencies between Phaeobacter phage MD18 and *P. inhibens* (right graph; *r *= 0.877; *P < *2.2 × 10^−16^) exhibit better correlation than Phaeobacter phage MD18 to Escherichia coli (left graph; *r *= 0.656; *P = *4.094 × 10^−9^). Blue dots indicate codons with corresponding tRNA genes within the phage genome. The dashed red line illustrates a perfect correlation.

We used tRNAscan-SE version 2.0 (24, 25) to identify tRNAs in the *Phaeobacter virus MD18* genome and found 32 tRNA genes (28 unique) (Table S2). These tRNA genes corresponded to codons which were significantly enriched in the *Phaeobacter virus MD18* genome compared to codons without a corresponding tRNA gene (Fig. 3A) (*P = *6.4 × 10^−5^, Wilcox rank sum test). Phage genomes frequently encode tRNA genes, which facilitate the translation of phage transcripts in host bacteria (26–28). Another assumption of phage genome evolution is that phage codon usage adapts to resemble that of the bacterial hosts (27, 28). Supporting this model, the relative codon preferences of all codons in *Phaeobacter virus MD18* genes were significantly positively correlated with codon usage in *P. inhibens* (*r *= 0.877; *P < *2.2 × 10^−16^) and less significantly correlated with a distantly related nonhost bacterium, Escherichia coli (*r = *0.656; *P = *4.094 × 10^−9^) (Fig. 3B).

10.1128/mSphere.00898-20.6 TABLE S2 tRNA genes identified in Phaeobacter phage MD18 genome. Download Table S2, XLSX file, 0.02 MB. Copyright © 2020 Urtecho et al. 2020 Urtecho et al. This content is distributed under the terms of the Creative Commons Attribution 4.0 International license.

### Identification of MD18-resistant Phaeobacter inhibens mutants in a barcoded transposon insertion mutant library.

**FIG 4:**
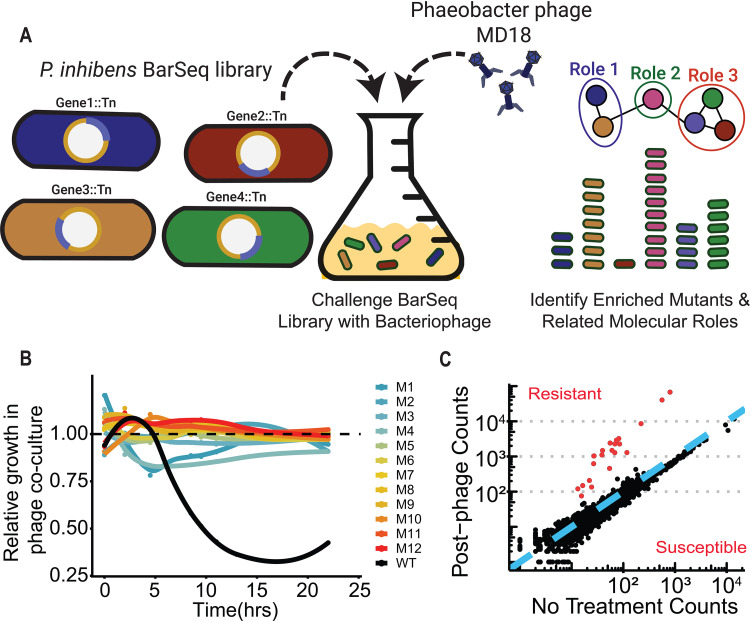
BarSeq screen identifies genetic loci that contribute to phage susceptibility. (A) Experimental scheme for BarSeq assay. After identification of a phage species capable of infecting *P. inhibens*, this phage was incubated with the BarSeq library and the barcodes of the survivors were sequenced. The relative abundance of the barcodes was compared to that of a parallel culture in which no phage was added, and the functional relationships between those genes were analyzed. (B) Twelve library variants that survived selection (M1 to M12; colored lines) were nearly entirely resistant to phage MD18, while wild-type (WT) growth was significantly hindered (black line). Relative growth is the ratio of the growth rate with phage compared to that without phage. (C) Comparison of BarSeq mutant counts grown with and without phage. Mutations in genes above the blue line (*y* = *x*) exhibited a relative increase in abundance following phage incubation, indicating enhanced resistance. Points in red are considered statistically significant and exhibited fitness scores 3 standard deviations less than or greater than the mean.

We sought to characterize the relationship between Phaeobacter phage MD18 and *P. inhibens* by identifying host gene products that confer susceptibility to infection. Recent work has used transposon insertion sequencing to rapidly perform reverse-genetic screens in hosts and identify genes that contribute to phage susceptibility (29–33). We selected for *P. inhibens* transposon insertion mutants with decreased susceptibility to phage by exposing a previously constructed barcoded transposon mutant library (32) to phage MD18 (Fig. 4A). This library consisted of 205,898 variants, each carrying a randomly barcoded transposon insertion mapped to 1 of 3,341 *P. inhibens* genes annotated using RAST (20, 21). To impose selection on this library, we incubated the exponentially growing library with Phaeobacter phage MD18 (1.5 × 10^8^ PFU) for 8 h, conditions which allowed for significantly delayed growth in wild-type *P. inhibens* (Fig. 1B). In parallel, we grew the same library without phage selection as a control comparison. After growth in triplicate of the phage-exposed and control cultures, we extracted DNA from all samples and sequenced barcodes in each population to measure the relative frequency of each *P. inhibens* transposon insertion mutant. Concurrently, we plated the postselection population on agar plates and isolated 12 individual clones at random. We found that all 12 isolates exhibited a marked increase in resistance to phage challenge (Fig. 4B).

**FIG 5:**
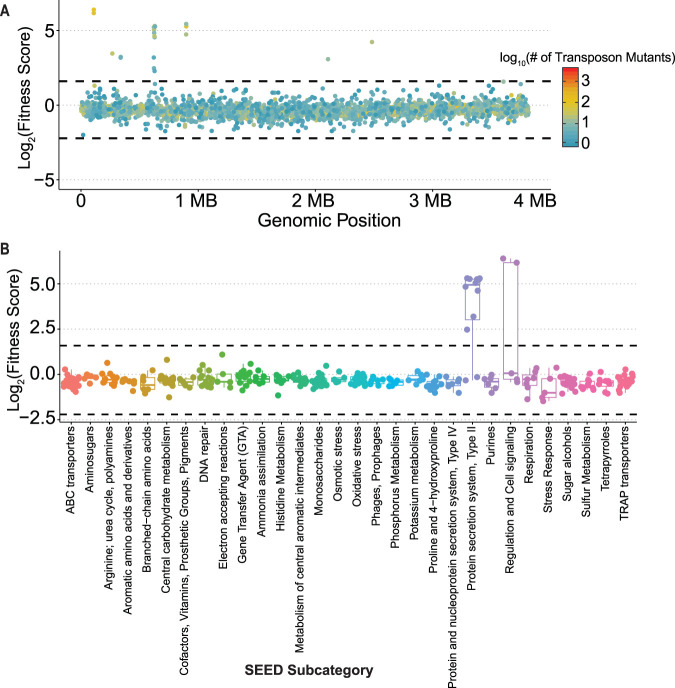
Enriched variants cluster by operon and Gene Ontology annotation. (A) Fitness of mutants across the *P. inhibens* genome. Each point represents an individual gene colored by the number of transposon insertion mutants tested for that gene. Dashed line indicates significance threshold, 3 standard deviations greater than the mean fitness score. Asterisks indicate locations of operons whose mutations exhibited significantly higher fitness scores. (B) SEED subcategory annotations indicate that genes encoding a type II secretion system and cell signaling pathways alter the fitness of *P. inhibens* in the context of phage MD18 infection. The dashed line indicates the significance threshold, set at 3 standard deviations greater than the mean fitness score. Colors indicate different SEED subcategories.

Using the relative abundances of barcodes present in the total cultures with and without phage exposure, we determined the fitness of all mutant strains within the BarSeq library. Of the 3,341 genes for which fitness values were determined, 22 exhibited significantly greater fitness scores when grown in the presence of phage (Fig. 4C and Fig. S4). Interestingly, these genes clustered within four primary operons, suggesting the presence of highly connected functions (Fig. 5A). Gene Ontology (GO) term analysis using SEED subsystem annotations (21) showed that these significant hits were enriched for genes encoding a type II secretion system involved in pilus formation (Fig. 5B). Other significant hits included the ChvI/ChvG two-component system and Fts cell division proteins (Table 1 and Table S3). We did not identify any genes with negative fitness scores, which would indicate a role in promoting phage resistance.

**TABLE 1:**
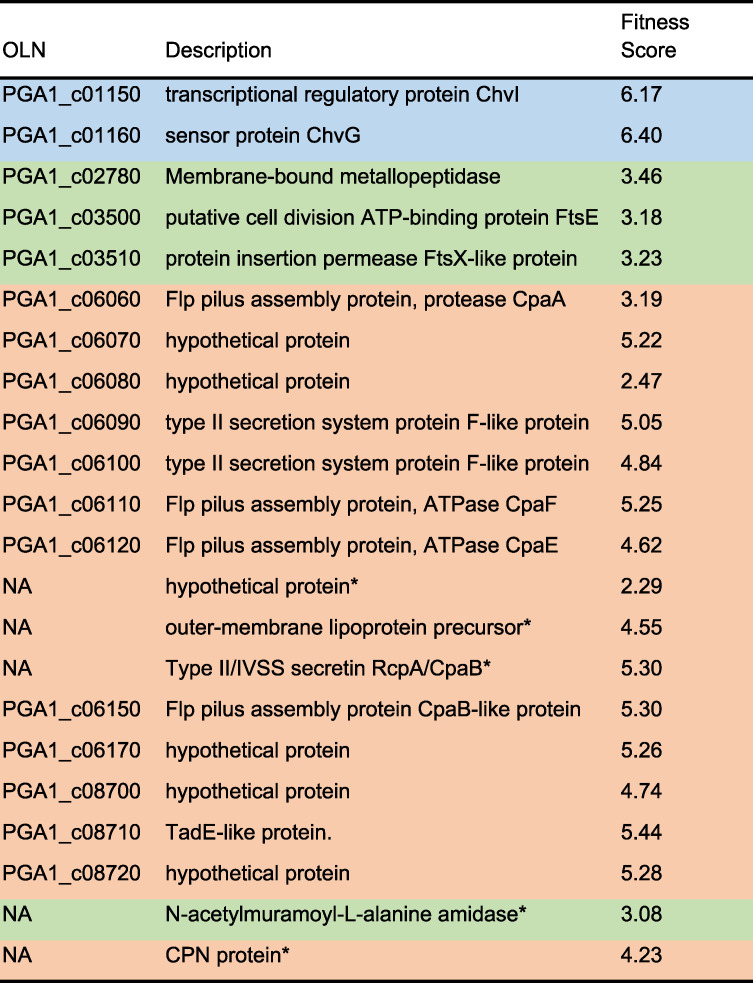
Genes identified in the BarSeq study^*a*^

a Genes are ordered by genomic position and colored according to STRING-assigned functional cluster (62). Ordered locus names (OLN) and open reading frame (ORF) descriptions were previously established (32). Asterisks indicate gene descriptions assigned using RAST. Fitness scores are presented as log_2_ values and calculated from the average barcode frequencies across three biological replicates.

10.1128/mSphere.00898-20.4 FIG S4 Distribution of gene fitness scores. Gene fitness scores for transposon insertion variants span a wide range. The red line indicates the significance threshold, set as 3 standard deviations greater than the bootstrapped mean of all observed fitness scores. Download FIG S4, PDF file, 0.1 MB. Copyright © 2020 Urtecho et al. 2020 Urtecho et al. This content is distributed under the terms of the Creative Commons Attribution 4.0 International license.

10.1128/mSphere.00898-20.7 TABLE S3 Genes identified from the transposon insertion sequencing analysis. Download Table S3, XLSX file, 0.02 MB. Copyright © 2020 Urtecho et al. 2020 Urtecho et al. This content is distributed under the terms of the Creative Commons Attribution 4.0 International license.

## DISCUSSION

In this study, we identified a novel lytic bacteriophage, Phaeobacter phage MD18, that infects *P. inhibens*. Based on the genomic analysis and morphological characteristics, we propose that MD18 is of the *Siphoviridae* morphotype. Furthermore, genomic and phylogenetic analyses assessing host range and a search of available metagenomes are all consistent with the conclusion that *Phaeobacter virus MD18* constitutes a novel viral species.

We also identified *P. inhibens* genes that confer susceptibility to MD18 using BarSeq. BarSeq has proven to be a promising platform for performing rapid reverse-genetic screens to dissect molecular pathways in bacteria (33, 34). In this experiment, large bacterial libraries of transposon insertion mutants containing unique DNA labels were generated and challenged with experimental conditions of interest. Sequencing and determination of the relative abundances of these genetic barcodes enable the discovery of genes modulating bacterial fitness under selected conditions. Our BarSeq analysis of the *P. inhibens-*MD18 interaction showed a clear enrichment for genes participating in type IV pilus assembly (35), a two-component regulatory system, and selected Fts proteins. Thus, our work has revealed several testable hypotheses for how phage MD18 interacts with *P. inhibens* and how phage-host interactions occur in nonmodel systems.

First is the hypothesis that Phaeobacter phage MD18 recognizes type IV pili as its receptors. These surface structures have been found to be common targets of phages (36–39), and it has been previously demonstrated that genetic deletion of host pilin subunits obviates phage binding and subsequent lysis (40). Interestingly, as type IV pili are widespread among bacteria genera, phages targeting these structures have been demonstrated to exhibit broad host-targeting capabilities in some cases (40). Although our study showed that MD18 did not exhibit lytic potential versus C. crescentus, it is possible that further exploration could identify other bacterial targets of MD18 that utilize type IV pili or related type II secretion systems. Several type IV pilus components exhibit significant sequence and structural similarities with bacterial type II secretion systems (41–43). The relationship between type IV pili and type II secretion systems is extensive, and it has even been shown that overexpression of certain type II components results in the formation of pili (35, 44). Future efforts could also utilize adsorption and motility assays to investigate a potential fitness trade-off for the *P. inhibens* host by losing the Tad pilus.

Second, mutating the *chvI*/*chvG* genes encoding a two-component system was the most potent inhibitor of phage susceptibility; however, it is unclear mechanistically how this two-component system plays a role in phage susceptibility and resistance. Previous studies have shown that mutants of the ChvI-ChvG two-component system lose membrane stability in Rhizobium leguminosarum, which could conceivably disrupt the formation of the pilus (45). Alternatively, this two-component system may play a role in regulation of the *pho* operon, as other studies have shown that bacteriophages may hijack this pathway to promote phosphorus uptake in the host, thereby increasing the rate of phage assembly (46). Finally, it is possible that the ChvI-ChvG two-component system directly regulates expression of the Tad pilus.

Several other genes that regulate cell division and assembly of the septum, including the *ftsE*-*ftsX* (47) heterodimer and the peptidoglycan remodeler *amiC* (48), were also implicated in altering phage susceptibility. Cells with mutations in these genes exhibit altered morphologies, which may also disrupt or alter pilus formation (49). Some of these mutations have been shown to lead to a filamentous cell morphology (47), a survival phenotype of many bacteria under stress that this study suggests may help in preventing phage predation. However, it has been shown in other cases that filamentation is associated with increased phage susceptibility (50). It is also possible that misregulated peptidoglycan remodeling physically inhibits injection of phage DNA, a process which is not fully elucidated for tailed phages (51).

The isolation, genome sequencing, and receptor identification of Phaeobacter phage MD18 enables future work to understand viral infections of *P. inhibens*. Understanding phage-host interactions in diverse nonmodel systems like *P. inhibens* will further our understanding of basic phage biology in ways that might increase our abilities to implement new phage technologies and therapies.

## MATERIALS AND METHODS

### Bacteriophage isolation and enrichment.

The Phaeobacter inhibens strain used in this work (DSM 17395) was originally isolated in Galicia, Spain, and is available through the Leibniz Institute DSMZ. *P. inhibens* DSM 17395 was grown in Difco marine broth 2216 liquid (MB), on plates supplemented with 1.5% agar, or in 0.5 to 0.7% top agar overlays at 30°C. The *P. inhibens*-infecting bacteriophage was isolated from Woods Hole Waterfront Park in Woods Hole, MA. A sterile 15-ml tube was loaded with a sample of seagrass and filled to 15 ml with seawater. After vigorous vortexing for 5 s, 5 ml of this sample was filtered using a 0.22-μm polyethersulfone (PES) syringe filter and divided into 1-ml aliquots. A single aliquot was incubated overnight at room temperature with 5 ml of log-phase (optical density at 600 nm [OD_600_] ∼ 0.3) *P. inhibens* in MB, centrifuged for 2 min at 2,000 × *g*, and filtered using a 0.22-μm PES syringe filter to obtain the enriched bacteriophage population. This enriched sample was used for all downstream experiments with no further purification. The presence of the isolated phage was confirmed by spotting onto 0.7% top agar inoculated with mid-exponential-phase *P. inhibens*. The isolated phage was named MD18 because it was isolated as part of the microbial diversity course at the Marine Biological Laboratory in Woods Hole, MA, during the summer of 2018.

### Bacteriophage characterization.

Top agar spotting and liquid growth assays were used to measure the concentration of PFU in the lysate and to characterize the plaque phenotype of the enriched phage MD18 isolate. Eight 10-fold dilutions of the enrichment were prepared by serial dilution in MB. To perform the spotting assay, 4 ml of MB with 0.5% molten agarose was cooled to ∼50°C before mixing with 1 ml of log-phase *P. inhibens*, and this mixture was spread evenly across a petri dish containing MB agar. Once solidified, 5 μl of each dilution was spotted and incubated for 16 h at 30°C. As a control to exclude the presence of a cellular predator, 10 μl of the undiluted phage stock was incubated with 1 μl of 2.0% chloroform for 1 h with shaking at 4°C before spotting on the same plate. Medium control was prepared by incubating 10 μl of MB with 1 μl of 2.0% chloroform for 1 h with shaking at 4°C. To perform the liquid growth assays in the presence of bacteriophage, a log-phase culture of *P. inhibens* was diluted 1:100 in MB and 190 μl was aliquoted into a 96-well Corning clear-bottom plate. To each well, 10 μl of each bacteriophage dilution or sterile MB was added (200 μl total per well) in triplicate. The plate was grown for 24 h at 30°C with shaking at 150 rpm, and the OD_600_ of each culture was monitored using a Promega GloMax Explorer multimode microplate reader.

### Transmission electron microscopy.

To prepare the bacteriophage for transmission electron microscopy, 10 μl of the enriched bacteriophage isolate lysate (∼10^10^ PFU/ml) was incubated with a glow-discharged Formvar-coated 200-mesh copper grid for 3 min before being washed three times with sterile 0.22-μm-filtered water. The sample was negatively stained by incubation in a 2% uranyl acetate solution under darkness for 1 min before undergoing another series of washes. For imaging host cells and phage MD18 together, 5 μl of the same phage preparation was preincubated with 5 μl of exponential-phase *P. inhibens* culture for 10 min and adhered to the grid and stained as described above. Samples were imaged using a Zeiss 10CA transmission electron microscope with help from the Marine Biological Laboratory Central Microscopy Core.

### Bacteriophage DNA sequencing, assembly, and annotation.

Phage MD18 was prepared by inoculating 100 μl of the phage enrichment (∼10^10^ PFU/ml) in 100 ml of mid-log-phase *P. inhibens* in MB. This culture was grown for 24 h with shaking before centrifugation and 0.22-μm filtration of the supernatant. This phage fraction was concentrated to ∼1 ml with Corning 30,000-molecular-weight-cutoff (MWCO) Spin-X UF 20 concentrators (Corning, NY).

DNA was extracted from this concentrated virus sample using a modified protocol with the Wizard genomic DNA (gDNA) purification kit (A1120; Promega, Madison, WI). Briefly, DNase I was added to 300 μl of phage sample at a final concentration of 1 μg/ml and incubated at room temperature for 1 h. To the virus sample, 480 μl of 50 mM EDTA and 900 μl of cell lysis solution were added, vortexed, and incubated for 30 min at 30°C. To this mixture, 600 μl of nucleus lysis solution was added, vortexed, and incubated for 5 min at 80°C. To this mixture, 3 μl of RNase A solution was added, vortexed, and incubated for 30 min at 37°C. To this mixture, 200 μl of protein precipitation solution was added, vortexed for 20 s, and incubated for 5 min on ice. Cellular debris was pelleted by centrifugation at 13,000 × *g* for 3 min, and the pellet was discarded. DNA was precipitated by the addition of 2.5 ml of isopropanol at –20°C and centrifugation at 13,000 × *g*, and the supernatant was discarded. The DNA pellet was washed in 600 μl of 95% ethanol at 20°C and centrifuged at 13,000 × *g*, and the supernatant was discarded. The DNA pellet was dried before resuspension in 100 μl of DNA rehydration solution.

Phaeobacter phage MD18 DNA was prepared for sequencing with the Nextera DNA Flex library prep kit and barcoded with the IDT for Illumina Nextera DNA UD Indexes (Illumina, San Diego, CA). The DNA was sequenced on an Illumina NovaSeq 6000 with a NovaSeq SP reagent kit, yielding 647,478 250-nucleotide (nt) read pairs. Reads were trimmed using Trimmomatic version 0.38, using the parameters SLIDINGWINDOW:4:15 LEADING:2 TRAILING:2 MINLEN:35, resulting in 623,304 reads. These reads were assembled using SPAdes (version 3.11.1) using default parameters. The primary assembled contig was submitted to the RAST (20, 21) online server (https://rast.nmpdr.org/rast.cgi) to identify putative genes and the tRNAscan-SE 2.0 (24, 25) online server to identify encoded tRNA genes (http://lowelab.ucsc.edu/tRNAscan-SE/). Genome feature tables were generated by converting GenBank (.gbk) files using the GB2sequin online server (52). The circular genome visualization was generated using Blast Ring Image Generator using default settings (61).

### Genomic comparisons to DSS3Ф8, CbK phage, and metagenomes.

Phylogenetic analysis of *Phaeobacter virus MD18* and potentially related species was performed using the Virus Classification and Tree Building Online Resource web server (VICTOR). C. crescentus phages were selected from reference 17 and are accessible through GenBank under the following accession numbers: phiCbK, JX100813; CcrMagneto, JX100812; CcrSwift, JX100809; CcrKarma, JX100811; CcrRogue, JX100814; and CcrColossus, JX100810. These accession numbers, along with those for Phaeobacter phage MD18 and Roseobacter phage DSS3Ф8 (accession no. KT870145), were submitted to the Virus Classification and Tree Building Online Resource web server (VICTOR, https://ggdc.dsmz.de/victor.php#).

VICTOR processed these genomes in the following manner. All pairwise comparisons of the nucleotide sequences were conducted using the genome BLAST distance phylogeny (GBDP) method (53) under settings recommended for prokaryotic viruses (54). The resulting intergenomic distances were used to infer a balanced minimum evolution tree with branch support via FASTME, including subtree pruning and regrafting postprocessing (55) for each of the formulas D0, D4, and D6. Branch support was inferred from 100 pseudobootstrap replicates each. Trees were rooted at the midpoint (56) and visualized with FigTree. Taxon boundaries at the species, genus, and family levels were estimated with the OPTSIL program (57), the recommended clustering thresholds (54), and an F value (fraction of links required for cluster fusion) of 0.5 (58).

Genome maps between Phaeobacter phage MD18 and Roseobacter phage DSS3Ф8 were generated in R (version 4.0.2) using the GenoPlotR package (version 0.8.9) (https://genoplotr.r-forge.r-project.org/). Genomes were aligned using NCBI BLAST (https://blast.ncbi.nlm.nih.gov/Blast.cgi) using discontiguous megablast, which is optimized for more dissimilar sequences. The hit table (text) file from the alignment along with the genome GenBank records (.gb) were used as input for GenoPlotR.

To determine whether MD18 had been previously identified in metagenome libraries, the MD18 genome was searched against a data set of 27,346 marine virome contigs assembled from 78 marine viromes (19). BLAST (59, 60) command line tools (version 2.10.1) were used to construct a BLAST database from the 27,346 viral contigs, and a *blastn* search of MD18 against this database was conducted using the default parameters (Gap Penalties: Existence: 0, Extension: 2.5).

### *P. inhibens* barcoded transposon insertion mutant library challenge.

The *P. inhibens* library was originally produced by Wetmore et al. (32) and generously provided by the Crosson lab. A 1-ml aliquot of this library was used to inoculate 30 ml of MB plus kanamycin (300 μg/μl; kanamycin is the antibiotic resistance marker carried by the transposon used to prepare the BarSeq library) and grown for 4 h before reaching an OD_600_ of ∼0.5. Three 1.5-ml aliquots of this culture were centrifuged at 2,000 × *g* for 2 min and the pellets frozen for later DNA preparation. To perform the bacteriophage challenge, 850 μl of the growing culture was used to inoculate six cultures of 30 ml of MB plus kanamycin (300 μg/μl), and 1.5 ml of 100-fold-diluted plaque-purified bacteriophage lysate (∼10^10^ PFU/ml) was added to three of these cultures. These six cultures were grown for 8 h, then 1 ml of each culture was centrifuged at 2,000 × *g* for 2 min, and the pellets were frozen for later DNA preparation.

### BarSeq sequencing library preparation.

In total, nine samples were processed for BarSeq. Three of these samples represented the original library, three represented the library grown without phage, and three more represented the library grown in the presence of phage. Genomic DNA from all samples was harvested using a Promega Maxwell RSC PureFood GMO and authentication kit. To prepare these samples for Illumina sequencing, sequencing primer sites, Illumina flow cell adapters, and sample indices were added using two successive PCRs. Primers used are described in Table S4. The first PCR amplified barcoded transposon sequences from the genomic insertion sites, and the second PCR added Illumina sequencing primer sites and flow cell adapter sequences. For the first PCR, 200 ng of each gDNA sample was amplified using Promega GoTaq G2 Hot Start polymerase with primers Barseq_P1_PCR1 and Barseq_P2_PCR1 (Table S4). The PCR was performed using the following conditions: 95°C for 5 min and 16 cycles of 95°C for 45 s, 59°C for 30 s, and 72°C for 1 min, followed by a final extension for 2 min at 72°C. The DNA from the 50 μl PCR products were each purified using 60 μl of Agencourt AMPure XP beads following the manufacturer’s protocols (Beckman Coulter; no. A63880). For the second PCR, 1 μl of each of the nine samples was amplified and indexed by a unique pair of primers, Barseq_P1_S51X and Barseq_P2_N72X, with GoTaq G2 Hot Start polymerase. PCR under the same conditions as before was performed for 10 cycles. The DNA from these PCRs was purified using 90 μl of Agencourt AMPure XP beads (Beckman Coulter; A63880) before being pooled in equimolar ratios and sequenced using an Illumina MiSeq paired-end 250-cycle kit multiplexed with other unrelated samples. Only the forward read of the sequencing run was used to simplify downstream sequence processing.

10.1128/mSphere.00898-20.8 TABLE S4 Primers used for BarSeq libraries (PCR 1 and PCR 2). PCR 1 added sequencing primers, whereas PCR 2 added indices and flow cell adapter sequences. Download Table S4, XLSX file, 0.01 MB. Copyright © 2020 Urtecho et al. 2020 Urtecho et al. This content is distributed under the terms of the Creative Commons Attribution 4.0 International license.

### Fitness calculation.

Transposon insertion mutant fitness was determined by the relative abundance of each barcode before and after challenge by bacteriophage compared to the relative abundance without bacteriophage. Barcode sequences were extracted from the raw reads and the number of each barcode in each sample was counted and normalized to reads per million (RPM). The *P. inhibens* DSM 17395 genome (GenBank no. CP002976.1) was annotated using the RAST (20, 21) online server (https://rast.nmpdr.org/), and barcodes were grouped by which RAST-identified gene they overlapped. Barcode transposon insertion sites were previously described by Wetmore et al. (32). All insertions were used, regardless of their positions within the gene. After grouping of barcodes by which genes they overlapped, genes were filtered out of our analysis if they were represented by fewer than 3 barcodes, as such samples are more susceptible to experimental noise. The fitness of each remaining gene was calculated as follows.

First, the gene enrichment scores with and without phage were calculated. This was determined by dividing the RPM-normalized counts of all barcode insertions overlapping each gene after treatment by the RPM-normalized counts before treatment.

$${\text{Enrichment}}_{\text{(treatment)}}=\frac{\sum \text{(overlapping barcode counts posttreatment)}}{\sum \text{(overlapping barcode counts, initial)}}$$

RPM-normalized counts were calculated from the mean of three biological replicates. Then, the fitness score of each gene disruption was calculated by dividing the sum of all enrichment scores within the phage treatment by the sum of all respective enrichment scores without phage added (see equations).

$$\text{Fitness score}={\text{log}}_{\text{2}}\left(\frac{{\text{enrichment}}_{\text{phage}}}{{\text{enrichment}}_{\text{no phage}}}\right)$$

Fitness scores were considered significant if they were greater or less than 3 standard deviations of the bootstrapped mean fitness score. All gene annotations were generated using RAST with default parameters and viewed using SEED to acquire Gene Ontology terms. To generate the gene network, amino acid sequences of the genes whose knockouts exhibited significantly greater fitness scores were uploaded as multiple sequences to the STRING online tool (https://string-db.org/) and searched against Phaeobacter inhibens DSM 17395. Default settings were utilized and online STRING tools were used to identify three gene clusters using K-means clustering.

### Data availability.

The Phaeobacter phage MD18 genome is available in GenBank (accession no. MT270409). *P. inhibens* transposon mutant fitness data are available on NCBI Gene Expression Omnibus (accession no. GSE148502). Code to analyze BarSeq data and generate figures is available on Github (https://github.com/gurtecho/PhaeobacterphageMD18).
