# Supplementary figures and images for: Discovering the Molecular Determinants of Phaeobacter inhibens Susceptibility to Phaeobacter Phage MD18
Source: mSphere. 2020 Nov 4;5(6):e00898-20. doi: 10.1128/mSphere.00898-20 (PMC7643831; doi:10.1128/mSphere.00898-20)

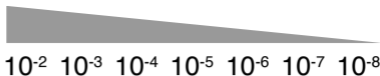

MD18

$\Phi$ CbK

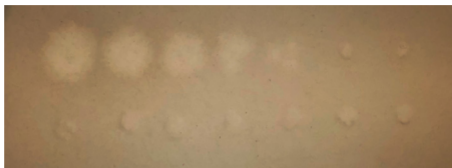

*P. inhibens* DSM17395

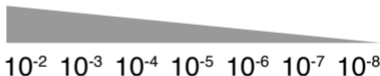

MD18

$\Phi$ CbK

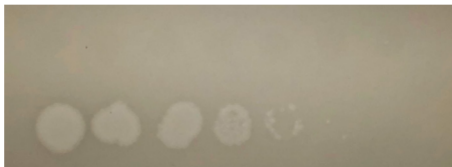

*C. crescentus* CB15

Supplement: FIG S1 [file mSphere.00898-20-sf001.pdf]

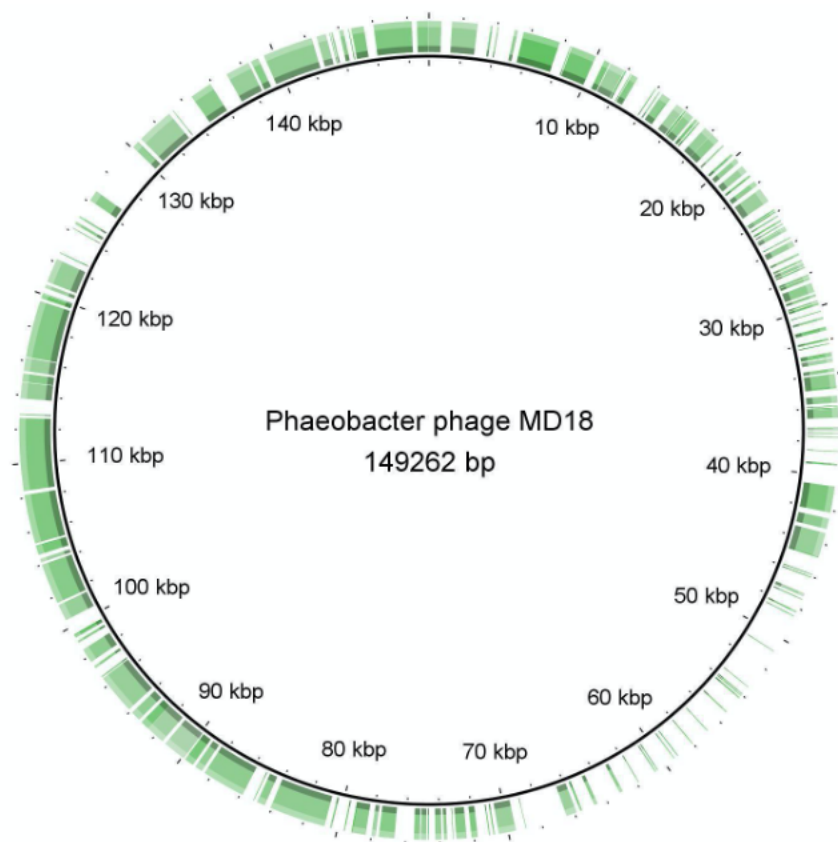

Supplement: FIG S2 [file mSphere.00898-20-sf002.pdf]

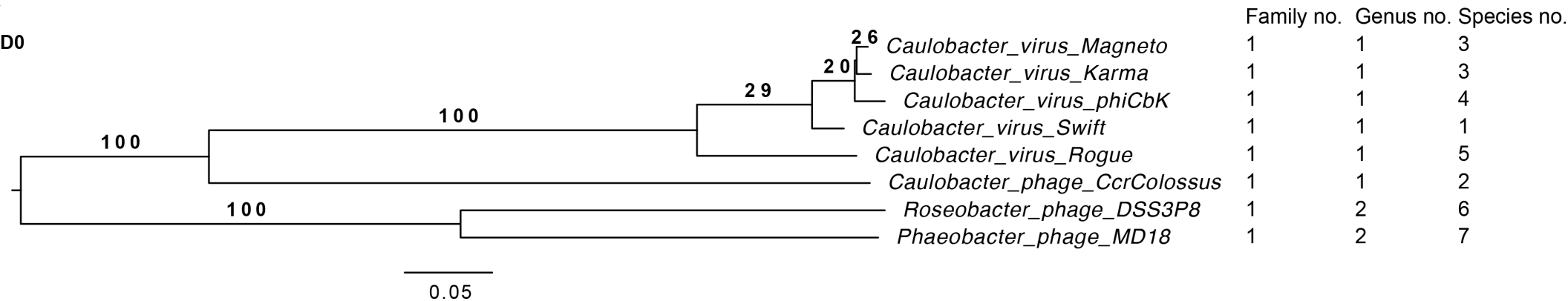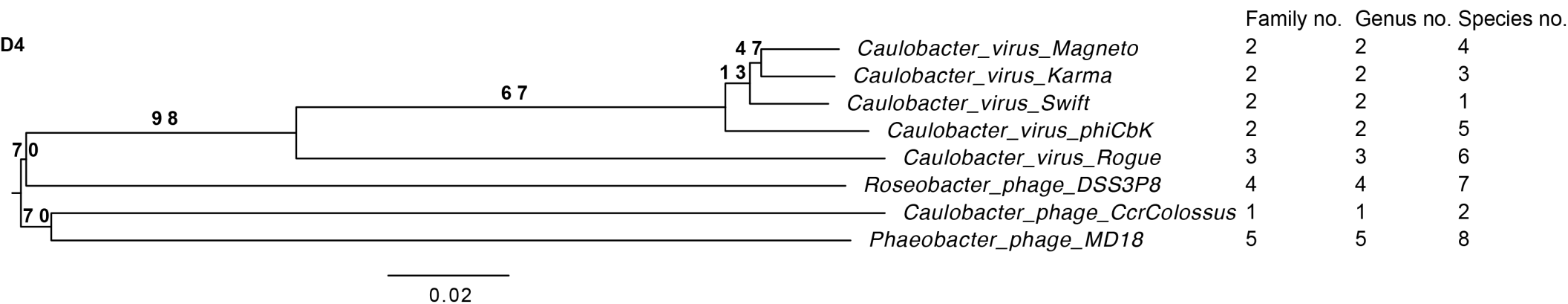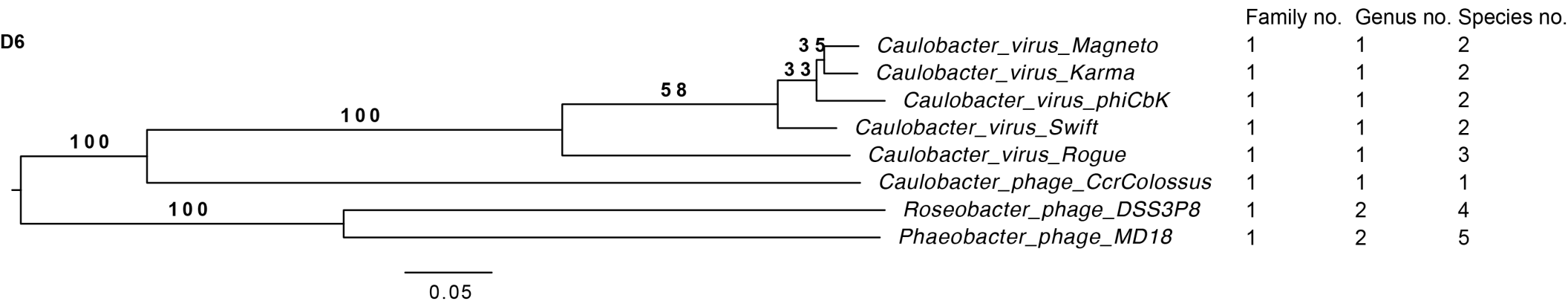

Supplement: FIG S3 [file mSphere.00898-20-sf003.pdf]

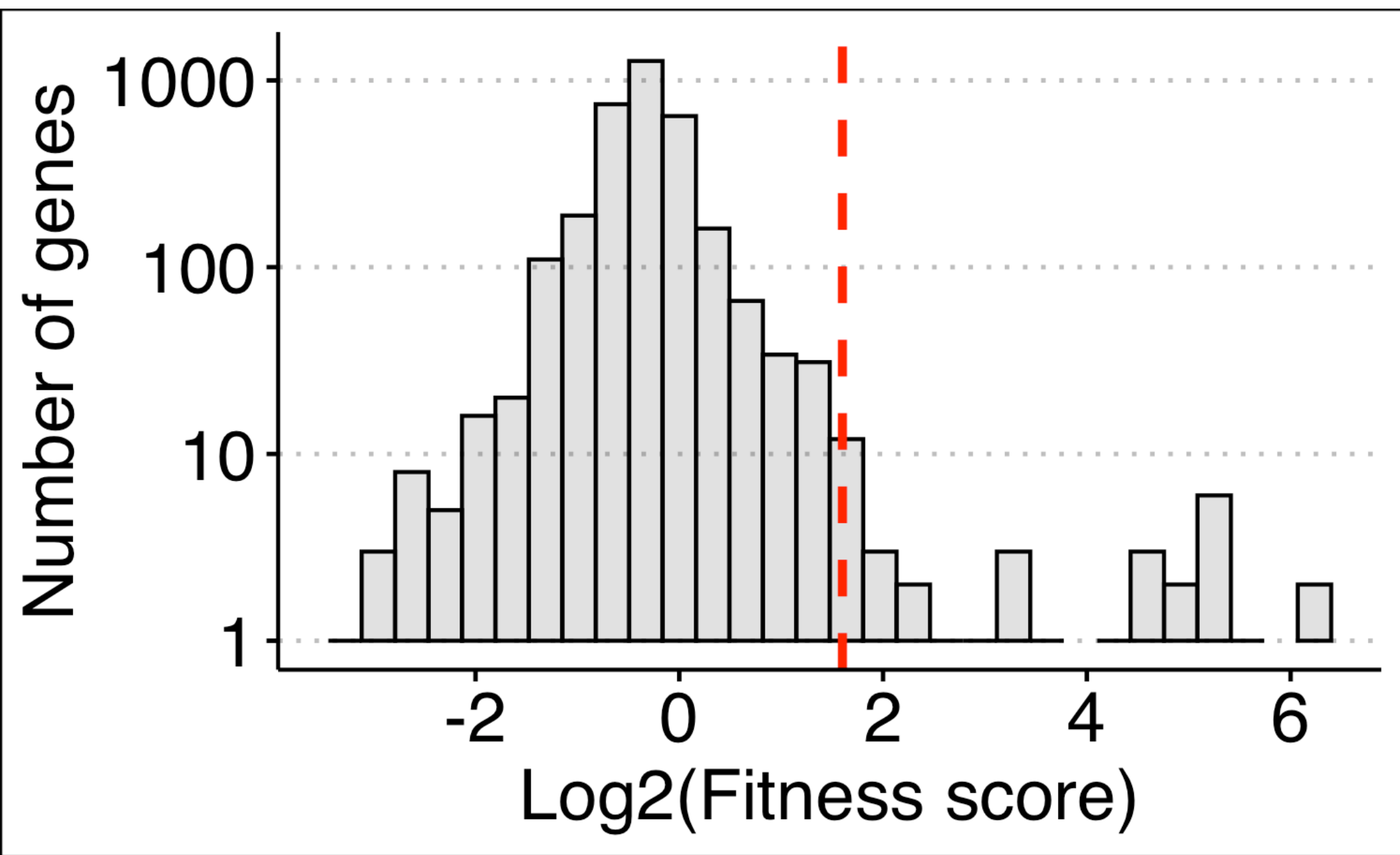

Supplement: FIG S4 [file mSphere.00898-20-sf004.pdf]
